# Molecular Evidence for Hybrid Origin and Phenotypic Variation of *Rosa* Section *Chinenses*

**DOI:** 10.3390/genes11090996

**Published:** 2020-08-25

**Authors:** Chenyang Yang, Yujie Ma, Bixuan Cheng, Lijun Zhou, Chao Yu, Le Luo, Huitang Pan, Qixiang Zhang

**Affiliations:** Beijing Key Laboratory of Ornamental Plants Germplasm Innovation & Molecular Breeding, National Engineering Research Center for Floriculture, Beijing Laboratory of Urban and Rural Ecological Environment, Key Laboratory of Genetics and Breeding in Forest Trees and Ornamental Plants of Ministry of Education, School of Landscape Architecture, Beijing Forestry University, Beijing 100083, China; cheny6622@foxmail.com (C.Y.); 13466780515@163.com (Y.M.); chengbixuan@126.com (B.C.); 13020021576@163.com (L.Z.); luole15804@163.com (L.L.); pantakki@126.com (H.P.); zqxbjfu@126.com (Q.Z.)

**Keywords:** *Rosa* sect. *Chinenses*, China rose, morphological traits, molecular marker, genetic diversity, phylogenetic analysis

## Abstract

*Rosa* sect. *Chinenses* (Rosaceae) is an important parent of modern rose that is widely distributed throughout China and plays an important role in breeding and molecular biological research. *R.* sect. *Chinenses* has variable morphological traits and mixed germplasm. However, the taxonomic status and genetic background of sect. *Chinenses* varieties remain unclear. In this study, we collected germplasm resources from sect. *Chinenses* varieties with different morphological traits. Simple sequence repeat (SSR) markers, chloroplast markers, and single copy nuclear markers were used to explore the genetic background of these germplasm resources. We described the origin of hybridization of rose germplasm resources by combining different molecular markers. The results showed that the flower and hip traits of different species in *R.* sect. *Chinenses* were significantly different. The SSR analysis showed that the two wild type varieties have different genetic backgrounds. The double petal varieties of *R.* sect. *Chinenses* could be hybrids of two wild type varieties. A phylogenetic analysis showed that the maternal inheritance of sect. *Chinenses* varieties had two different origins. To some extent, variation in the morphological traits of double petal species of *R.* sect. *Chinenses* reflects the influence of cultivation process. This study emphasizes that different genetic markers vary in their characteristics. Therefore, analyzing different genetic markers in could provide an insight into highly heterozygous species.

## 1. Introduction

Modern rose is the largest group of ornamental plants in the world, with more than 30,000 cultivars [1]. *Rosa* sect. *Chinenses* (Rosaceae) represents one of the hybrid parents of modern rose. *Rosa* contains approximately 200 species, more than 95% of which belong to the subgenus *Rosa*. Species in the subgenus *Rosa* can be divided into 10 sections, *R.* sects *Pimpinellifoliae, Rosa, Cinnamomeae, Synstylae, Chinenses (Indicae), Banksianae, Laevigatae, Bracteatae, Caninae, and Carolinae* [2]. A total of 82 *Rosa* species can be found in China, with species in *R*. sect. *Chinenses* endemic to China. *R*. sect. *Chinenses* contains three species: *Rosa chinensis* Jacq, *Rosa odorata* (Andr.) Sweet, and *Rosa lucidissima* Lévl. Three varieties of *R. chinensis* are currently recognized: *R. chinensis* var. *chinensis*, var. *spontanea* (Rehd. et Wils.) Yu et Ku, var. *semperflorens* (Curbs) Koehne. While four varieties of *R. odorata* are recognized: *R. odorata* var. *odorata*, var. *gigantea* (Crep.) Rehd. et Wils., var. *pseudlindica* (Lindl.) Rehd., var. *erubescens* (Focke) Yu et Ku. *R. chinensis* var. *spontanea* and *R. odorata* var. *gigantea* are considered as original species, both of which have single petal. [3]. Roses in China were first described in the Han Dynasty [4] and then spread all over China, leading to the breeding of diverse cultivars. In *China’s Ancient Rose*, 180 old garden roses from ancient China were recorded, including 29 different *R. chinensis* varieties and 22 different *R. odorata* varieties, which have single or double petals. Varieties with double petals are considered as more cultivated [1]. Molecular studies have shown that the varieties with double petal of *R*. sect. *Chinenses* might be hybrids of *R. odorata* var. *gigantea* and *R. chinensis*. *Rosa multiflora* might be involved in multiple hybridization events of *R. chinensis* cultivars [5].

With unique ornamental characters, China roses became important plant materials in breeding and molecular studies. However, the description of double petal varieties of *R. chinensis* and *R. odorata* is not sufficiently clear in the *Flora of China* [3]. A large number of putative natural or artificial hybrid varieties have been found in the wild, and the genetic relationship among these species has not yet been completely elucidated. A previous study suggested that these species were transitional populations generated from breeding [6] or the hybrid offspring of wild and cultivated populations.

The phenotypic variation of *Rosa* species is highly complex and results from the interaction between genes and their environment. Morphological markers have allowed for the study of genetic diversity and taxonomy of some *Rosa* species [7,8,9]. Compared with morphological markers, molecular markers are not affected by environmental factors, and they can directly reflect the differences and similarities between genetic materials. Diverse molecular markers are currently available, but different markers have distinct functions. To date, hundreds of simple sequence repeats (SSRs) had been developed for *Rosa* [10,11], and these have been applied to the classification of wild and cultivated roses [12,13]. In addition, single-copy nuclear genes (SCGs) can also be effectively used to research the characteristics of parental inheritance, orthologs, and high genetic polymorphism of *Rosa*. *GAPDH* is one of the SCGs and it is the most commonly used in the genus *Rosa* [14,15] that is more suitable for taxonomic categories above class [16]. With the release of the rose genome [17,18], SCGs are being more widely applied [19]. Chloroplast genes have been shown to be more conserved than nuclear genes [20]. And chloroplast genes are maternally inherited [21], while nuclear genes are inherited from both parents. Thus, combining the two types of markers could be used to identify and infer the maternal contributor to hybrid roses [5,9]. Integrating the advantages of different genetic markers could provide further insight into the genetic diversity of *Rosa* species. In this study, a series of *R*. sect. *Chinenses* germplasm resources was collected. The genetic diversity of these resources was analyzed using different genetic markers, and the genetic background and taxonomic status of these materials were clarified.

## 2. Materials and Methods

### 2.1. Experimental Materials

**Figure 1:**
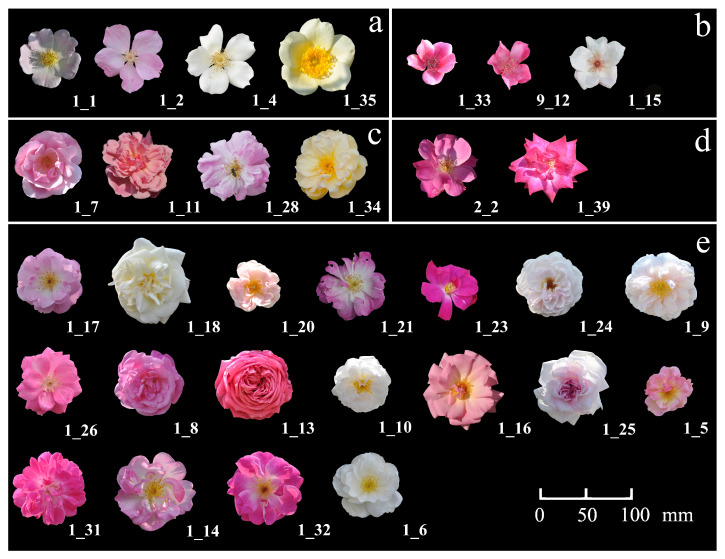
Variation of rose accession flowers: (**a**) *R. odorata* var. *gigantea* (**b**) *R. chinensis* var. *spontanea* (**c**) *R. odorata* var. *odorata* (**d**) *R. chinensis* var. *chinensis* (**e**) *R.* sect. *Chinenses* complex. While (**a**,**b**) are wild type rose accessions, (**c**–**e**) are the cultivated types of rose accessions.

In this study, 31 rose accessions were used, including eight *R. odorata* (Andr.) Sweet, five *R. chinensis* Jacq and 18 *R*. sect. *Chinenses* varieties with transition phenotypes (Table 1). Most plant materials were collected from Yunnan Province, with the exception of single petal varieties (Figure 1). The collections were cultivated at Kunming Yang Chinese Rose Gardening Co., Ltd. (Kunming, China), and traits were observed for successive years to confirm the stability of phenotypic characters and determine whether morphological differences existed among the materials. Among the phenotypic characters, different accessions of *R*. sect. *Chinenses* displayed significant differences in several quantitative traits. Flower and hip-related traits in rose are affected by both environment and genotype [22,23,24]. Therefore, the materials used in this study were cultivated in an open field and had grown in the same environment for many years. This allowed the traits to stabilize. Thus, the phenotypic variations described in this study were primarily derived from the genotype.

Because the *Flora of China* lacks a detailed description of double petal varieties of *R. chinensis* and *R. odorata* [1], they have not been subordinately classified. Varieties with unclear taxonomic status but similar to *R. odorata* or *R. chinensis* are difined as *R*. sect. *Chinenses* complex. *R. odorata* var. *gigantea* and *R. chinensis* var. *spontanea* with single petal character were the original species of *R*. sect. *Chinenses*. The combination of *R. odorata* var. *gigantea* and *R. chinensis* was designated the wild type [3], and the rest of the sect. *Chinenses* accessions have characteristics of double petal. They were denoted as cultivated type [1].

### 2.2. Measurement and Analysis of Phenotypic Traits

Thirteen floral and hip phenotypic traits of *R*. sect. *Chinenses* were measured (Table 2). A single-factor analysis of variance (ANOVA) was used to compare the differences of traits among species. A nonparametric Kolmogorov–Smirnov test (K-S test) and median test were used to analyze the significance of phenotypic differences among species. A principal component analysis (PCA) was conducted using FactoMineR Package [25] implemented in R v. 3.6.3.

### 2.3. DNA Extraction, Amplification, and Sequencing

Total DNA was extracted from the young leaves of annual branches collected from plant material described above. The leaf material was dried with silica gel and stored at room temperature. Total DNA was extracted using the Plant Genome Extraction Kit (DP320) from Tiangen Biotech Co., Ltd. (Beijing, China) and was used as the template for downstream analysis. PCR amplification of the DNA template was performed using primers for three types of markers, including SSRs, chloroplast DNA, and SCGs. SSRs and chloroplast primers were adopted and screened from previous studies [26,27,28,29]. SCGs were screened from the rose genome sequence. First, this procedure used 959 amino acid sequences of *Arabidopsis* SCGs that were shared among *Arabidopsis*, *Populus*, *Vitis*, and *Oryza* [30]. These sequences were then compared against the *R. chinensis* ‘Old Blush’ genome [18] using TBLASTN [31]. Sequences with an identity > 60% and only one hit were selected as the candidate SCG. 363 SCGs were selected. Amplification primers were designed using two genes from each chromosome among the 363 SCGs. Each amplified fragment was established to be between 600 bp and 1500 bp and contained at least one exon region. Finally, four SCG markers were screened for this experiment (Table 3).

The PCR reagent from the 2 × PCR Master Mix from Beijing BioDee Biotechnology Co., Ltd. (Beijing, China) was used. PCR amplifications were performed in 20 μL reactions containing the following: 1 μL DNA template, 1 μL upstream primer, 1 μL downstream primer, and 10 μL 2 × PCR Master Mix, and were brought to volume using ddH2O. The PCR amplification program was as follows: pre-denaturation at 95 °C for 2 min, 30 cycles of denaturation at 95 °C for 30 s, annealing for 30 s, extension at 72 °C for 60 s, and a final extension at 72 °C for 5 min. The annealing temperature was set according to the primers used. The primer information is listed in Table S1. The amplification products of SSR markers were detected using a 3730XL DNA analyzer (Applied Biosystems, ThermoFisher Scientific, Waltham, MA, USA), and the electrophoretic results were assessed by GeneMapper software [32] to identify the size of each fragment. The amplification products of SCG markers and chloroplast gene markers were directly sequenced. The resulting sequences were assembled, and the mismatch sites were manually corrected.

### 2.4. Analysis of Genetic Diversity

The results of SSR analysis were statistically analyzed using Genealex v. 6.5 [33], and the number of different alleles (Na) and polymorphic information content (PIC) of each variety were calculated. Heterozygous excess was detected using Bottleneck v. 1.2, with the TPM model, 80% of Stepwise Mutation Model (SMM), and 10% of Infinite Alleles Model (IAM) [34]. The genetic structure was analyzed using STRUCTURE v2.3.4 [35], and eight independent simulations at each level of genetic clustering (K; for K = 2–7) were performed. A Markov chain Monte Carlo (MCMC) analysis for each simulation repeats 1,200,000 times after a burn-in period of 200,000. The best K was estimated using the online tool Structure Harvester [36], and a sampling analysis for each K was conducted using CLUMPP [37].

Multiple sequence alignment of SCGs and chloroplast markers was performed using MAFFT [38], and the results were inputted into DnaSP v. 6.12 [39] for a statistical analysis of nucleotide polymorphisms and haplotype. A phylogenetic analysis was conducted on chloroplast gene markers and SCG markers using PhyloSuite [40]. For SCG markers, the conserved region of each gene was obtained by a BLAST search against ‘Old Blush’ genome, and the non-conserved region was manually deleted. Chloroplast marker sequences were compared against the chloroplast genomes of *R. odorata* var. *gigantea* (KF753637) [41], *R. chinensis* var. *spontanea* (MG523859) [42], ‘Old Blush’ (CM009590) [18], R. multiflora (MG727863) [43], and R. lucieae (MG727864) [43], and the reference sequence were aligned with sequenced results. After the sequences were assembled and partitioned, the models of sequence evolution were tested by PartitionFinder [44] under the corrected Akaike’s Information Criterion (AICc). The phylogenetic analysis used the Bayesian analysis implemented by MrBayes [45]. The program was run with MCMC chain generations = 2,000,000, sampling frequency = 100, burn-in fraction = 0.25, the number of chains = 4 and the number of runs = 2. In addition, the frequency of chloroplast haplotypes was counted, and the haplotype network was constructed using the software PopART 1.7 [46] based on the Median Joining method [47].

## 3. Results

### 3.1. Variance Analysis of Phenotypic Traits

Most quantitative traits differed significantly among varieties (Table 2). The results of the K-S test and median test showed significant differences in nine traits among different species. Multiple comparisons revealed that the hip of the wild type accessions was significantly larger than those of the cultivated type accessions. The flower diameter and sepal size of *R. odorata* were significantly larger than those of *R. chinensis*. The *R*. sect. *Chinenses* complex had a mid-sized flower diameter and sepal size. Additionally, the pistil and peduncle length differed among varieties.

**Figure 2:**
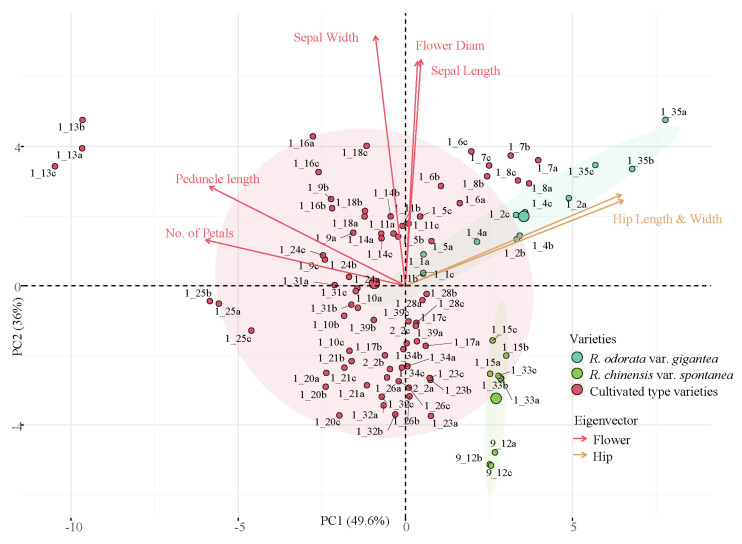
Principal component analysis of *R*. sect. *Chinenses* morphological characters. Letters behind the accessions numbers indicate the repeated experiments for each accessions. While the larger point indicate the center of the ellipse.

The PCA was performed, and the data was graphed for phenotypic traits (Figure 2). The first two principal components explained 95.6% of phenotypic variation. Principal component one (PC1) (49.6%) primarily explained the effects of hip length and width, peduncle length, petal number, and length of pistil and stamen. Principal component two (PC2) (36%) primarily explained the effects of sepal length and width and flower diameter. The figure showed that PC2 could effectively distinguish the phenotypic differences between *R. odorata* var. *gigantea* and *R. chinensis* var. *spontanea*, while the distribution of phenotypes of cultivated type partially overlapped with wild type.

### 3.2. Genetic Diversity of SSR Markers

A total of 221 allelic variations were detected in 15 SSR loci in the materials tested, and each locus was polymorphic. The number of alleles ranged from seven to 26, with an average of 14.73 for each locus. The PIC ranged from 0.873 to 0.576, with an average of 0.804. They showed a high degree of genetic polymorphism in *R*. sect. *Chinenses*. These SSR loci could effectively distinguish different individuals.

The bottleneck effect was detected on three combinations of all accessions, the wild type accessions and the cultivated accessions (Table 4). The results showed that under the IAM evolutionary model, the combination of double petal accessions displayed an excess of heterozygosity at a significance level of 0.05, indicating they had experienced a bottleneck effect. Under the TPM evolutionary model, this combination also displayed a bottleneck effect at a significance level of 0.1. Vegetative propagation in cultivation, multiple origins, and extensive hybridization could cause the bottleneck effect [6].

**Figure 3:**
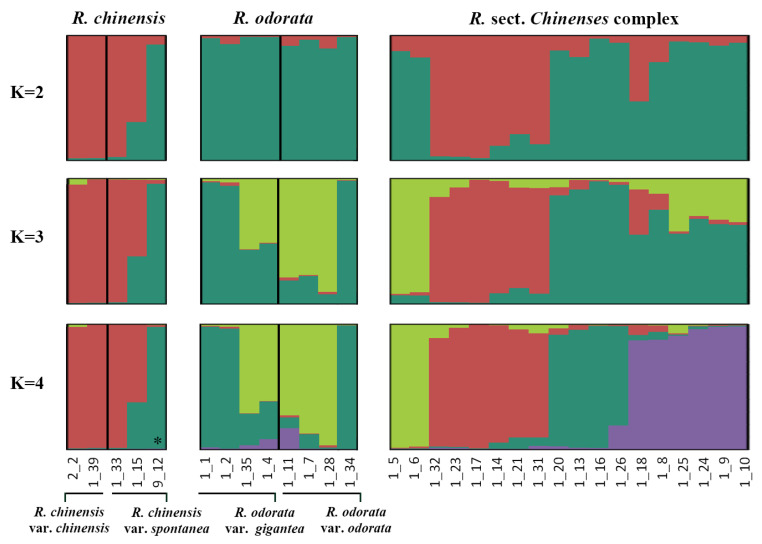
Genetic structure of *R*. sect. *Chinenses*.

**Figure 4:**
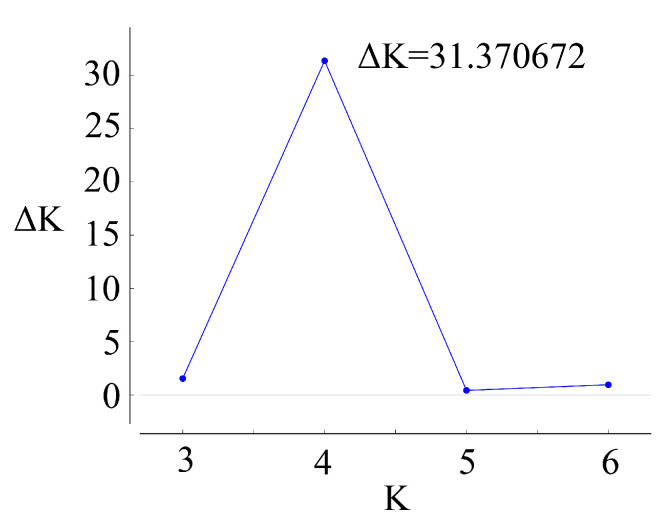
The value of delta K (averaged across 8 runs) obtained from STRUCTURE software [35].

The genetic structure analysis (Figure 3) identified that the best K was four (Figure 4). The genetic structure was graphed according to the biological classification of the accessions. The results showed that when K = 2, *R. chinensis* and *R. odorata* were composed of different ancestral components, while the *R*. sect. *Chinenses* complex contained two components. When K = 3, *R. odorata* was composed of two different ancestral groups, while the *R*. sect. *Chinenses* complex contained three components. When K = 4, accessions with a higher degree of heterozygosity in the *R*. sect. *Chinenses* complex formed a new component. In addition, an accession of *R. chinensis* var. *spontanea* (labeled with * in the figure) with European provenance showed different components.

### 3.3. Screening of SCGs

**Figure 5:**
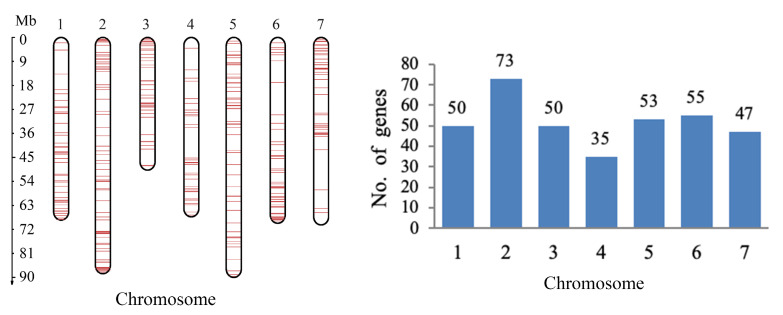
Distribution of the 363 studied single-copy nuclear genes (SCGs) in the ’Old Blush’ genome.

A total of 363 SCGs were screened from the ‘Old Blush’ genome (Figure 5), and two genes on each linkage group were selected for primer design and fragment amplification. Four SCG markers with high amplification efficiency and sequencing accuracy were used in the following study.

### 3.4. Phylogenetic Analysis

The genetic diversity of each locus was calculated (Table 3). Due to different genetic patterns between chloroplast and nuclear genes, the two types of markers were separately assembled, aligned, and analyzed phylogenetically.

**Figure 6:**
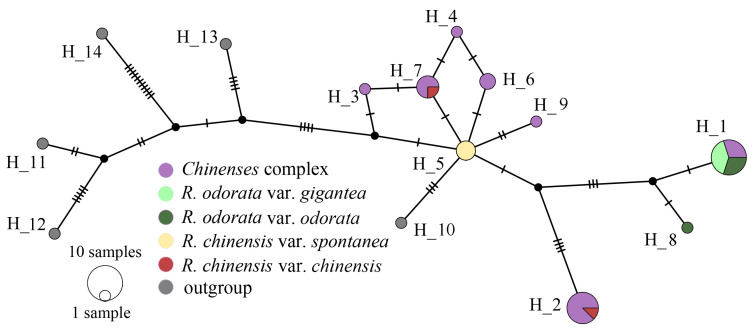
Haplotype network of *R*. sect. *Chinenses* based on chloroplast markers.

Based on the results of haplotype analysis on chloroplast genes, all accessions could be divided into 14 haplotypes (H1 to H14) (Figure 6), in which H1 to H9 were the haplotypes of sect. *Chinenses*, H10 to H14 were the outgroup haplotypes. No common ancestor was found for H10 from *R. multiflora* between other outgroups. *R. odorata* var. *gigantea* and *R. chinensis* var. *spontanea* accessions belong to H1 and H5 respectively. H1 and H8 were identified in the cultivated type of *R. odorata*, and H8 and H1 were derived from the very close common missing haplotype. H2, H3, H4, H6, H7, and H9 of the remaining cultivated type were mutated from H5 (*R. chinensis* var. *spontanea*).

**Figure 7:**
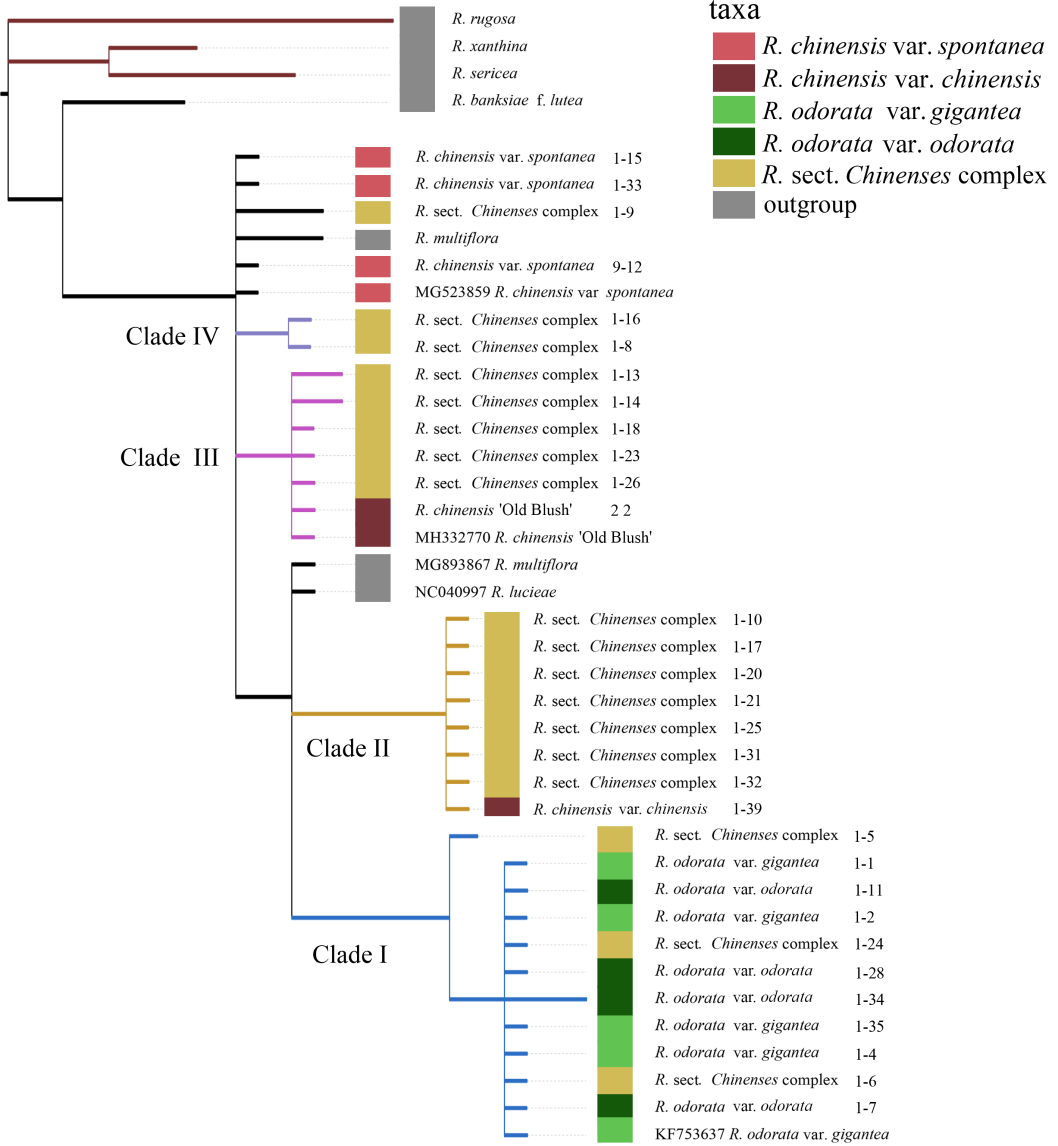
Phylogenetic analysis of *R*. sect. *Chinenses* based on chloroplast markers.

The phylogenetic analysis of chloroplast sequences revealed four main clades (Figure 7). Clade I contained all *R. odorata* accessions and reference sequences. Clades II and III were composed of the *R*. sect. *Chinenses* complex. *R. chinensis* var. *spontanea* formed several independent branches, indicating that these accessions were more ancestral. *R. multiflora* (section *Synstylae*) was not clustered in the outgroup clade but in different positions within the in-group.

**Figure 8:**
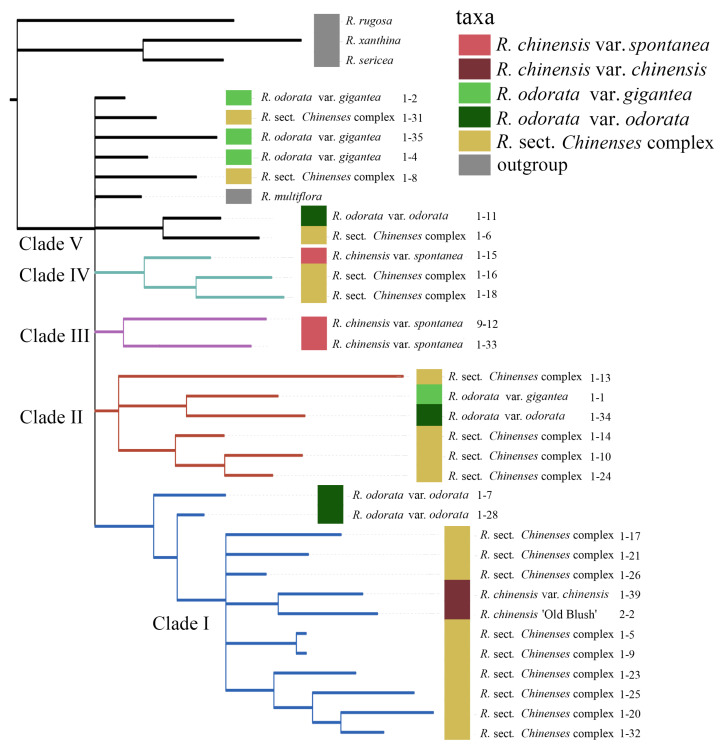
Phylogenetic analysis of *R*. sect. *Chinenses* based on single copy nuclear gene markers.

The phylogenetic analysis based on SCGs resolved five main clades (Figure 8). Clade I contained the *R*. sect. *Chinenses* complex, two *R. chinensis* accessions, and two *R. odorata* accessions. Clade II contained two *R. odorata* accessions and some accessions from the *R*. sect. *Chinenses* complex. Clade III contained *R. chinensis* var. *spontanea* and *R. lucieae*. Another *R. chinensis* var. *spontanea* accession was located in clade IV together with two accessions from the *R*. sect. *Chinenses* complex.

## 4. Discussion

### 4.1. Origin of Cultivation of the China Rose

The genetic diversity in rose encompasses two sources: wild species and cultivars [48]. *R. odorata* var. *gigantea* and *R. chinensis* var. *spontanea* are the wild types of *R*. sect. *Chinenses*. China roses have undergone thousands of years of cultivation. The flower type of China roses is simlar to that of cultivated type varieties. The cultivated types has been predicted to include the transition varieties produced in the breeding process. Alternatively, they could be the new varieties generated by hybridization between cultivated rose and wild species. Some cultivars have been identified as potential hybrid varieties [5]. The genetic structure analysis showed that *R. chinensis* and *R. odorata* possessed distinct genetic backgrounds, while the *R*. sect. *Chinenses* complex was heterozygous with two species, suggesting that these accessions may be hybrids of *R. chinensis* and *R. odorata*. Owing to the influence of geographical isolation of the Red River Fault Zone, two evolutionary significant units were identified in *R. odorata* var. *gigantea* [49]. In this study, the geographical distribution of *R. odorata* var. *gigantea* was consistent with the description above. No correlation between the genetic background and geographical location was found in cultivated type of *R. odorata*.

Chloroplast markers are maternally inherited and are not affected by hybridization factors. The haplotype analysis suggested that six out of the seven haplotypes of cultivated type varieties were derived from *R. chinensis* var. *spontanea*, and only one haplotype was shared with *R. odorata* var. *gigantea*. These findings indicated that most of the accessions have maternal genetic background similar to *R. chinensis* var. *spontanea*. The haplotypes of cultivated type *R. odorata* were derived from *R. odorata* var. *gigantea*, but nuclear gene markers showed that the genetic background was inconsistent with that of *R. odorata* var. *gigantea*.

Previously published phylogenetic studies of *Rosa* showed that *R*. sects *Synstylae* and *Chinenses* were closely related. The two sections were often embedded together [15,50,51,52] or became sister clades [19], and *R*. sect. *Synstylae* may have participated in the formation of *R*. sect. *Chinenses* varieties [5,18]. In this study, *R. multiflora* from *Synstylae* did not form a clade with other outgroups but appeared in the sect. *Chinenses* clade. This indicated that *R. multiflora* may be involved in the hybridization of sect. *Chinenses* varieties. However, owing to the limitation of experimental materials, it was difficult to explain the relationship between *R. multiflora* and *R*. sect. *Chinenses* in more detail. Future studies should include more materials from *R*. sect. *Synstylae* to further clarify the phylogenetic relationships.

### 4.2. Variation of Phenotypic Characters of Cultivated Type Varieties

The analysis of phenotypic characters suggested significant differences in phenotypes between the wild type and cultivated types. A comparison of differences among species revealed that the flower diameter of *R. odorata* was significantly greater than that of *R. chinensis*. For the comparison of differences between wild type and cultivated types, the diameter of flower of cultivated types was found to be larger than that of its maternal parent, *R. chinensis* var. *spontanea*. A previous study showed that the inheritance of flower diameter was directly related to the number of petals and is affected by the additive effect of dominant genes [53]. The hip of the cultivated varieties was significantly smaller than that of the two wild type roses, indicating that the hip size of *R*. sect. *Chinenses* accessions decreased over the course of cultivation.

We found that no trait for fasciculate inflorescence existed in the wild type varieties, whereas a trait for fasciculate inflorescence existed in the cultivated type varieties. These results suggested that the phenotypes of fasciculate inflorescence appeared gradually with the continuous occurrence of artificial or natural hybridization. The pattern of genetic inheritance of rose inflorescences is currently unknown and two independent developmental pathways were related to it. Diverse variations in inflorescences have been observed in *Rosa* species, such as *R. multiflora* with panicles. While these wild roses might have participated in the hybridization of *R. chinensis* cultivars [5], this trait may be derived from other *Rosa* species.

### 4.3. Role of Rose Germplasm Resources

Germplasm resources are the basis for ornamental plant breeding. Although *R*. sect. *Chinenses* have contributed significantly to modern rose breeding throughout the world, a large amount of germplasm resources has not been fully explored and utilized. *R*. sect. *Chinenses* are being widely cultivated in many places across China. These germplasm resources are rich in ornamental traits and have substantial potential for molecular breeding research. However, owing to the complex genetic backgrounds and traits partially shared by different species, their taxonomic status is difficult to determine, and they were previously divided into different infraspecific levels [3,54]. The rose germplasm resources used in this study had the same characteristics. They were predicted to be transitional varieties produced during the breeding process or new varieties generated by further hybridization between cultivated and wild type accessions. These materials were of substantial significance to re-visit the breeding process of rose, the study of rose omics, the investigation of ornamental traits, the identification of functional genes, and hybrid breeding.

## Figures and Tables

**Table 1:** Classification of rose accessions.

Accession Number	Collection Locality	Taxonomic Assignation	Section	Haplotype Assignation
1_1	Dali, Yunnan, China	*Rosa odorata* var. *gigantea*	*Chinenses*	H1
1_2	Dali, Yunnan, China	*R. odorata* var. *gigantea*	*Chinenses*	H1
1_4	Kunming, Yunnan, China	*R. odorata* var. *gigantea*	*Chinenses*	H1
1_35	Kunming, Yunnan, China	*R. odorata* var. *gigantea*	*Chinenses*	H1
1_7	Dali, Yunnan, China	*R. odorata* var. *odorata*	*Chinenses*	H1
1_28	Dali, Yunnan, China	*R. odorata* var. *odorata*	*Chinenses*	H1
1_11	LIjiang, Yunnan, China	*R. odorata* var. *odorata*	*Chinenses*	H1
1_34	LIjiang, Yunnan, China	*R. odorata* var. *odorata*	*Chinenses*	H9
1_33	-	*Rosa chinensis* var. *spontanea*	*Chinenses*	H5
1_15	Wenchuan, Sichuan, China	*R. chinensis* var. *spontanea*	*Chinenses*	H5
9_12	-	*R. chinensis* var. *spontanea*	*Chinenses*	H5
1_39	-	*R. chinensis* var. *chinensis*	*Chinenses*	H2
2_2	-	*R. chinensis* ’Old Blush’	*Chinenses*	H7
1_5	LIjiang, Yunnan, China	*Rosa* sect. *Chinenses* complex	*Chinenses*	H1
1_6	LIjiang, Yunnan, China	*R*. sect. *Chinenses* complex	*Chinenses*	H1
1_8	Kunming, Yunnan, China	*R*. sect. *Chinenses* complex	*Chinenses*	H6
1_9	Kunming, Yunnan, China	*R*. sect. *Chinenses* complex	*Chinenses*	H10
1_10	Kunming, Yunnan, China	*R*. sect. *Chinenses* complex	*Chinenses*	H2
1_13	Puer, Yunnan, China	*R*. sect. *Chinenses* complex	*Chinenses*	H3
1_14	Puer, Yunnan, China	*R*. sect. *Chinenses* complex	*Chinenses*	H4
1_16	LIjiang, Yunnan, China	*R*. sect. *Chinenses* complex	*Chinenses*	H6
1_17	LIjiang, Yunnan, China	*R*. sect. *Chinenses* complex	*Chinenses*	H2
1_18	Tengchong, Yunnan, China	*R*. sect. *Chinenses* complex	*Chinenses*	H7
1_20	Kunming, Yunnan, China	*R*. sect. *Chinenses* complex	*Chinenses*	H2
1_21	-	*R*. sect. *Chinenses* complex	*Chinenses*	H2
1_23	-	*R*. sect. *Chinenses* complex	*Chinenses*	H7
1_24	Dali, Yunnan, China	*R*. sect. *Chinenses* complex	*Chinenses*	H1
1_25	Dali, Yunnan, China	*R*. sect. *Chinenses* complex	*Chinenses*	H2
1_26	-	*R*. sect. *Chinenses* complex	*Chinenses*	H7
1_31	Dali, Yunnan, China	*R*. sect. *Chinenses* complex	*Chinenses*	H2
1_32	-	*R*. sect. *Chinenses* complex	*Chinenses*	H2
outgroup				
24	-	*Rosa multiflora*	*Synstylae*	H10
9	-	*Rosa rugosa*	*Cinnamomeae*	H14
36	-	*Rosa banksiae* f. *lutea*	*Banksianae*	H13
57	-	*Rosa sericea*	*Pimpinellifoliae*	H12
10	-	*Rosa xanthina*	*Pimpinellifoliae*	H11

**Table 2:** A single-factor analysis of variance (ANOVA) of quantitative traits of *R.* sect. *Chinenses*.

Traits	Median (mm)					*p* Value	
	*R. odorata* var. *gigantea*	*R. odorata* var. *odorata*	*R. chinensis* var. *spontanea*	*R. chinensis* var. *chinensis*	Sect. *Chinenses* Complex	Median Test	K-S Test
Hip length	21.70 ${}^{a}$	17.44 ${}^{ab}$	20.22 ${}^{a}$	14.74 ${}^{bc}$	13.20 ${}^{c}$	<0.0001	<0.0001
Hip width	21.93 ${}^{a}$	16.16 ${}^{a}$	19.06 ${}^{a}$	14.11 ${}^{b}$	12.94 ${}^{c}$	<0.0001	<0.0001
Peduncle length	11.32 ${}^{a}$	14.25 ${}^{ab}$	5.03 ${}^{c}$	18.67 ${}^{bd}$	24.65 ${}^{d}$	<0.0001	<0.0001
Sepal length	27.90 ${}^{a}$	17.52 ${}^{b}$	16.56 ${}^{b}$	18.56 ${}^{b}$	22.42 ${}^{ab}$	0.0021	0.0292
Sepal width	6.25 ${}^{a}$	6.10 ${}^{a}$	4.02 ${}^{b}$	4.78 ${}^{a}$	6.71 ${}^{a}$	0.0038	<0.0001
Flower diameter	80.05 ${}^{a}$	78.32 ${}^{a}$	56.97 ${}^{b}$	62.14 ${}^{bc}$	67.14 ${}^{c}$	<0.0001	<0.0001
Number of petals	5 ${}^{a}$	29 ${}^{b}$	5 ${}^{a}$	22 ${}^{b}$	28 ${}^{b}$	<0.0001	<0.0001
Pistil length	3.24 ${}^{ab}$	3.43 ${}^{ab}$	2.35 ${}^{a}$	3.80 ${}^{b}$	5.19 ${}^{c}$	<0.0001	<0.0001
Stamen length	7.88	7.06	5.84	5.40	6.43	0.1080	0.0091

Letters in the table represent ANOVA homogeneous subsets based on a median test. The significance level is 0.05.

**Table 3:** Genetic diversity of gene markers.

Locus Name	RefSeq	Length	Polymorphism Sites	Nucleotide Polymorphism	Favorite Model
*matK*	-	533 bp	10 (1.9%)	0.00378	K81uf + G
*atpB-rbcL*	-	594 bp	9 (1.5%)	0.00171	K81uf + G
*trnL-trnF*	-	961 bp	26 (2.7%)	0.00412	K81uf + G
*ZIP4*	LOC112169932	720 bp	53 (7.4%)	0.01008	HKY + I + G
*AP5*	LOC112197902	1371 bp	44 (3.2%)	0.00127	HKY + I + G
*SQD1*	LOC112170325	767 bp	35 (4.6%)	0.00557	HKY + I + G
*ALG8*	LOC112182822	622 bp	47 (7.6%)	0.01134	HKY + I + G

**Table 4:** Bottleneck effect of different combinations.

Test Combination	*p* Value of Wilcoxon Sign-Rank Test	
	IAM	TPM
All accessions	0.06372	0.33026
Cultivated type	0.03534	0.08325
Wild type	0.84692	0.10700

IAM Infinite allele model, TPM Two-phase mutation model [34].

## Supplementary Materials

The following are available online at https://www.mdpi.com/2073-4425/11/9/996/s1, Table S1: Primer information of molecular markers.
