# Supplementary material for: Molecular Evidence for Hybrid Origin and Phenotypic Variation of Rosa Section Chinenses
Source: Genes (Basel). 2020 Aug 25;11(9):996. doi: 10.3390/genes11090996 (PMC7564265; doi:10.3390/genes11090996)
Supplement: Supplementary file 1 [file genes-11-00996-s001.pdf]

genes

MDPI

Table S1. Primer information of molecular markers.

**Locus name**	type	Annealing temperature	Location	Product length		Primer sequence (5'-3')
**336**	SSR	59.9	chrom4	159-191 bp	Forward	CAAACGAAACCCTCTGCTTC
					Reverse	GACGATGCATTTGGTGTGAC
353	SSR	59.7	chrom5	202-216 bp	Forward	CGCCCTAGTCTCCTCTCTCTC
					Reverse	CTCAAGCTGAAGCTCGGAGT
373	SSR	58.9	chrom6	90-114 bp	Forward	ACAAACTTCGCGATTCCTCT
					Reverse	AGTTCCAGACGTTGGAGTGC
397	SSR	59.9	chrom4	213-256 bp	Forward	GGCCTAGCAAAGCAACAAAC
					Reverse	AGTGGAGGGCAGTCTCTGAA
405	SSR	59.9	chrom7	265-295 bp	Forward	CAGCGAAAAGAACAAGGACC
					Reverse	CAGAAGCTAATAAATTAACAATCACCA
490	SSR	60	chrom6	120-139 bp	Forward	ACAACCAACCCAAGAACTCG
					Reverse	TCCCAGCTTCAGTCTCACCT
521	SSR	60.5	chrom6	214-257 bp	Forward	GTTCCAGCAGCACTCCAAGT
					Reverse	AGAGGGGATTAGCTGCACTG
541	SSR	59.7	chrom6	234-263 bp	Forward	CTACTCCAATGTCCGCTTCC
					Reverse	GTTGGAGAAGAAGCCGTGAG
593	SSR	60.1	chrom3	268-292 bp	Forward	TAACCAGGTCCTCACGAAGG
					Reverse	AACAAATCCCCCAGGATAGG
625	SSR	59	chrom7	121-196 bp	Forward	CGCGTCTCTCACATCTCAAA
					Reverse	AAGATCTTCTCTCCGGCCTT
648	SSR	60.5	chrom6	164-188 bp	Forward	CCTAAAGCTTAAGCCCCCAA
					Reverse	GCAATAGACTTGGCAGCCTC
682	SSR	59.7	chrom3	193-219 bp	Forward	TTCTTGAGCTAAAAGTGCATCG
					Reverse	CAGATCCAAACCGAACCCTA
CL2996	SSR	55	chrom2	170-182 bp	Forward	GCCACCATAGCCAGAGACAT
					Reverse	AGAAGAAGTTGACGACAGGGAC
H23017	SSR	55	chrom1	187-235 bp	Forward	ACACCAAGCAAACCAAAACC
					Reverse	AGCACGAAAACCGAGAGAGA

**Locus name**	type	Annealing temperature	Location	Product length		Primer sequence (5'-3')
Rw22A3	SSR	52.9	chrom6	108-162 bp	Forward	AGAGAATTGAAAAGGGCAAG
					Reverse	GAGCAAGCAAGACACTGTAA
ZIP4	SCG	57.1°C	Chrom6	842 bp	Forward	TCCTTTTTCATCATTTTGCACCTGT
					Reverse	GCTGAGGTCTGTTTACGCCA
AP5	SCG	56.4°C	Chrom4	1500 bp	Forward	TCGCTAACTGGCAGCATAGG
					Reverse	TCCTTATCTCTGGGTTGCTGT
SQD1	SCG	57.1°C	Chrom6	832 bp	Forward	TGGTCATTGGTGGAGATGGC
					Reverse	GCAGCAAACCGACTTTGTGT
ALG8	SCG	58°C	Chrom1	709 bp	Forward	AGCTATGGTGGTTCTTCGCC
					Reverse	GCCAGAGAGATTACCTGCCC
matK	chloroplast DNA	56.4°C	chloroplast	650 bp	Forward	CTATATCCACTTATCTTTCAGGAGT
					Reverse	GATTGGTTACGGGAGAAAAAG
trnL-trnF	chloroplast DNA	54.1°C	chloroplast	1000 bp	Forward	CGAAATCGGTAGACGCTACG
					Reverse	ATTTGAACTGGTGACACGAG
atpB-rbcL	chloroplast DNA	46.4°C	chloroplast	600 bp	Forward	GAAGTAGTAGGATTGATTCTC
					Reverse	CATCATTATTGTATACTCTTTC
